# Sustained effects of developmental exposure to inorganic arsenic on hepatic *gsto2* expression and mating success in zebrafish

**DOI:** 10.1242/bio.060094

**Published:** 2024-03-06

**Authors:** Abigail Ama Koomson, Patrice Delaney, Nouf Khan, Kirsten C. Sadler

**Affiliations:** Program in Biology, New York University Abu Dhabi, Saadiyat Island, United Arab Emirates

**Keywords:** Zebrafish, Arsenic, Liver, Developmental origins of health and disease

## Abstract

The impacts of exposure to the pervasive environmental toxicant, inorganic arsenic (iAs), on human and fish health are well characterized and several lines of evidence suggest that some impacts can manifest years after exposure cessation. Using a developmental exposure protocol whereby zebrafish embryos were exposed to 0.5 and 1.5 mM iAs from 4–120 hours post fertilization (hpf) and then removed, we investigated the sustained effects of iAs on gene expression in the liver, survival, reproductive success, and susceptibility to iAs toxicity in the subsequent generation. Persistent exposure to iAs during development had substantial effects on the hepatic transcriptome, with 23% of all expressed genes significantly changed following developmental exposure. The *gsto2* gene is involved in iAs metabolism and this gene was significantly downregulated in female livers 9 months after iAs was removed. Developmental exposure to 1.5 mM iAs, but not 0.5 mM, decreased survival by over 50% at 3 months of age. Adults that were developmentally exposed to 0.5 mM iAs had reduced mating success, but their offspring had no differences in observable aspects of development or their susceptibility to iAs toxicity. This demonstrates that developmental exposure of zebrafish to iAs reduces long-term survival, reproductive success and causes sustained changes to *gsto2* expression in the liver.

## Teaser

**Summary:** This work investigates the long-term effects of developmental exposure to inorganic arsenic (iAs) in zebrafish. Fish that were exposed to iAs during development had increased mortality as adults, sustained changes to *gsto2* expression in female livers and decreased mating success.

## INTRODUCTION

Exposure to inorganic arsenic (iAs) causes a wide range of adverse health outcomes in humans, fish and wildlife (Ahmed et al., 2013; Amuno et al., 2020; Bears et al., 2006; Boyle et al., 2008; Han et al., 2019). iAs is a ubiquitous element naturally present in the bedrock that leaches into water that is used by humans and is also a habitat for fish and other aquatic species, which are immersed in contaminated water and thus highly vulnerable to the toxic effects. Toxicant exposure overall, and iAs in particular, impacts fish health, reproductive success and suitability for contaminated fish as a food source. Thus, iAs has major impacts for food security and the global ecosystem.

Chronic exposure to iAs in humans causes skin lesions, cardiovascular diseases, respiratory disorders, neurodevelopmental issues, cancer and liver disease (Chen et al., 2023; Frediani et al., 2018; Martinez et al., 2011; Moon et al., 2017; Sanchez et al., 2018; Smeester and Fry, 2018; Xie et al., 2023). We and others have shown that the effects of iAs on the mammalian liver also occur during acute exposure of zebrafish (Bambino et al., 2018; Delaney et al., 2023 preprint, 2020; Hallauer et al., 2016; Lam et al., 2006; Li et al., 2016; Sarkar et al., 2017). Importantly, the health consequences of iAs exposure can occur even after the exposure has terminated (Thomas, 2013; Young et al., 2018). Data from human and animal studies show that iAs is associated with developmental origins of health and disease (Smeester and Fry, 2018; Young et al., 2018). For instance, in humans, exposure to iAs *in utero* or during childhood increases the risk of several diseases (Farzan et al., 2013), including neurological and cognitive defects, developmental delays (Raqib et al., 2009; Rosado et al., 2007; Tolins et al., 2014; Wasserman et al., 2007), and risk of infection (Farzan et al., 2013; Rahman et al., 2011; Raqib et al., 2009). This is illustrated by a case study in Chile showing that early life arsenic exposures increased the rate of liver cancer mortality (Liaw et al., 2008), and these effects persisted in adulthood, with individuals more likely to develop lung and bladder cancer even 40 years after iAs was removed from water sources (Steinmaus et al., 2013). Mouse studies demonstrate that developmental exposure to iAs increases cancer in the liver and other organs (Waalkes et al., 2006) and zebrafish exposed to iAs produced offspring that were smaller than controls (Hallauer et al., 2016), had locomotor defects, decreased survival as adults (Abu Bakar et al., 2022), and decreased motor activity (Valles et al., 2020). Thus, while the health effects of acute exposure to iAs are serious, there is further concern about long-term effects of exposure to both humans and wildlife.

Several studies have investigated the acute and chronic effects of iAs in fish, but few have investigated the long-term or generational effects of developmental exposure. Zebrafish are a widely used model for studying environmental toxicants (Aluru, 2017; Bambino and Chu, 2017; Gamse and Gorelick, 2016), offering many advantages due to their external fertilization, accessibility to large sample sizes, outbred backgrounds, rapid development and transparency of embryos and larvae. Similar to mammals, the zebrafish liver is the primary site of iAs toxicity in zebrafish, and exposure of both larvae and adults causes hepatotoxicity (Bambino et al., 2018; Carlson et al., 2013; Carlson and Van Beneden, 2014; Lam et al., 2006; Li et al., 2016; Ramdas Nair et al., 2022; Sarkar et al., 2017; Yang et al., 2019). As in mammals, iAs toxicity is attributed to cell stress responses, including oxidative and proteostatic stress (Delaney et al., 2020; Fuse et al., 2016; Lam et al., 2006; Ramdas Nair et al., 2022; Xu et al., 2013). We optimized experimental approaches for using zebrafish in toxicology studies (Ramdas Nair et al., 2021) and used this model to study iAs toxicity. We discovered that, like humans, iAs accumulates in the zebrafish liver and that the liver is a major source of iAs toxicity (Bambino et al., 2018; Delaney et al., 2023 preprint) and identified developmental time points between 4–5 days post fertilization (dpf), which coincides with liver development, as the prime window of susceptibility to iAs exposure (Delaney et al., 2020). Other studies demonstrated that exposure to iAs during critical developmental stages negatively impact survival, growth, behavior, and has heritable, detrimental effects (Abu Bakar et al., 2022; Hallauer et al., 2016; Valles et al., 2020). Specifically, developmental exposure to iAs (5–72 hpf) reduced long-term survival and affected swimming behavior, increased anxiety behaviors and impaired learning in adults (Abu Bakar et al., 2022). Other work has shown that progeny from adults exposed to low doses of iAs for 6 months were small (Hallauer et al., 2016) and that ancestral iAs was associated with reduced motor function in subsequent generations (Valles et al., 2020). While DNA methylation and histone modifications have been proposed to transmit the long-term effects of many toxicants, including iAs (Pilsner et al., 2012; Valles et al., 2020), the epigenetic basis of immediate, latent and heritable consequences of developmental iAs-exposure has not been elucidated in any species.

Here, we use developmental exposure of iAs in zebrafish to investigate the effects on survival, reproductive outcomes, liver gene expression and DNA methylation in exposed animals and on embryonic development and arsenic susceptibility of offspring from adults that were exposed developmentally. Animals that were exposed to high levels of iAs during development had reduced survival, and exposure to lower levels had sustained gene expression changes in the liver and reduced mating success. We found no effects on bulk DNA methylation in exposed animals, and the embryonic development and arsenic toxicity was unchanged in the offspring of adults that were developmentally exposed. This suggests that developmental exposure of iAs in zebrafish embryos has significant health and reproductive effects.

## RESULTS

### Zebrafish liver gene expression recovers from acute (4–5 dpf) iAs exposure

**Fig. 1.**
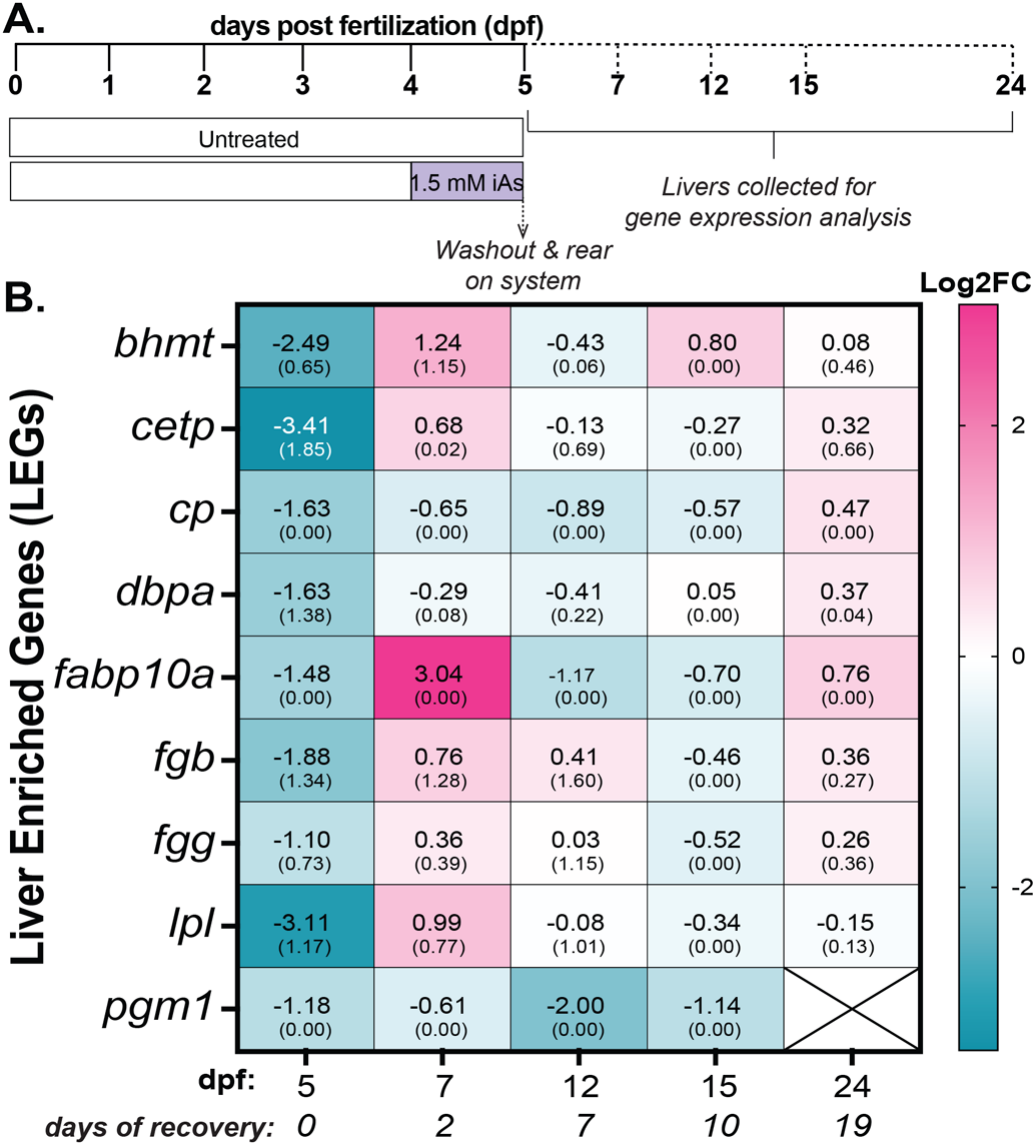
**Arsenic responsive genes recover in the liver by 10 days following washout from acute exposure to 1.5 mM iAs**. (A) Treatment scheme of acute treatment (4–5 dpf) to 1.5 mM iAs. At 5 dpf, iAs was washed-out (WO) and zebrafish were reared in the aquaculture system. At the indicated time points, livers were dissected, pooled, and analyzed for gene expression. (B) Heat map of the average log2 fold change (Log2FC) of the expression of genes analyzed in 5–7 livers following iAs WO. All genes have data from two clutches except for *cp, fabp10a*, and *pgm1* which have data from one clutch for both control and treated samples. The average values from one or two clutches are shown (top) with the standard deviation (SD) shown in parentheses.

To determine whether acute (4–5 dpf) exposure to iAs causes long-lasting effects on the liver after iAs was removed, we developed a treatment scheme whereby wild-type (WT) sibling zebrafish larvae were exposed to 0 or 1.5 mM iAs [the approximate lethal concentration at which 50% of larvae die (LC_50_)] and then washed to remove all residual iAs (Fig. 1A). Larvae were reared to 24 dpf, encompassing a total time of 19 days post iAs wash out. Livers were dissected from treated and untreated controls at 5, 7, 12, 15, and 24 dpf (0, 2, 7, 10 and 19 days post iAs-washout respectively) and 5–7 livers were pooled for gene expression analysis.

A subset of genes reflecting liver function (*bhmt, cetp, cp, dbpa, fabp10a, fgb, fgg, lpl and pgm1*) that we previously reported as downregulated following this treatment protocol (Delaney et al., 2023 preprint; Ramdas Nair et al., 2022) were analyzed by quantitative real-time PCR (qRT-PCR) at multiple time points after iAs was removed. Immediately following iAs exposure, these genes were downregulated (Fig. 1B), reflecting hepatotoxicity. At 2 days post-iAs removal, six of these genes increased expression, and by 10 days, expression of all genes returned to baseline levels (Fig. 1B; Table S1). This demonstrates that livers in zebrafish recover from acute iAs-toxicity within 10 days of removal.

### Developmental exposure to iAs induces widespread gene expression changes in the liver and sustained reduction of liver size

**Fig. 2.**
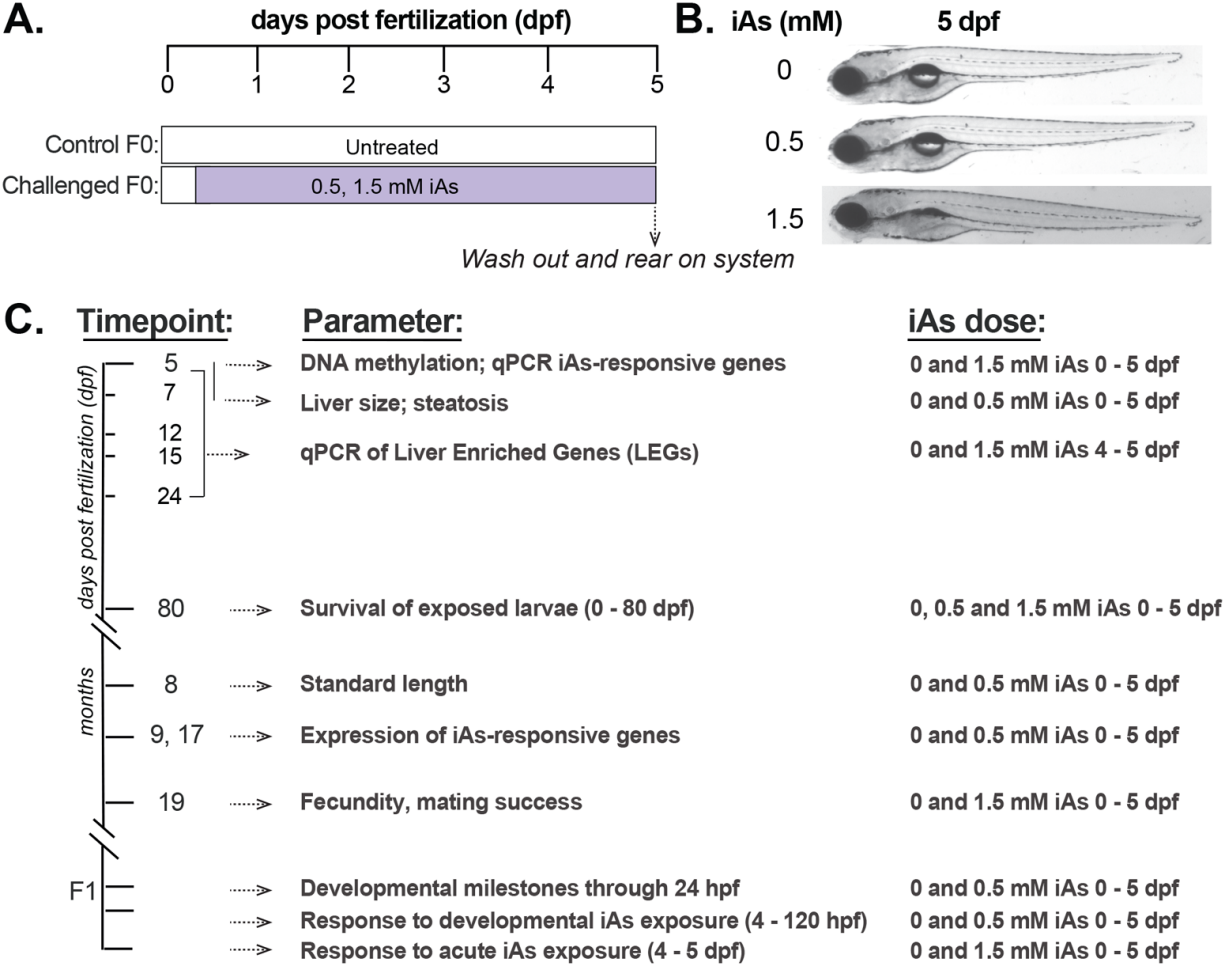
**Treatment scheme to evaluate long-term effects of developmental iAs exposure.** (A) Treatment scheme of arsenic exposure where F0 embryos were exposed to 0, 0.5 or 1.5 mM of iAs from 4–120 hpf and then washed out and reared on system. (B) Representative photos of 120 hpf wild-type zebrafish exposed to 0, 0.5 or 1.5 mM iAs from 4–120 hpf. (C) The timeframe of sample collection and parameters assessed.

Since we observed a relatively rapid recovery of the zebrafish liver to acute iAs exposure, we reasoned that prolonged developmental exposure could have more potent effects after removal. A previously optimized protocol in which zebrafish were exposed to iAs during major developmental processes including late gastrulation and organogenesis, (4–120 hpf) caused multiple developmental defects, with a lethal concentration 50 (LC_50_) of 1.5 mM (Bambino et al., 2018), while other studies showed that 0.5 mM iAs did not impact the survival or development of zebrafish embryos (Delaney et al., 2020; Li et al., 2009). We therefore used a protocol whereby zebrafish embryos were exposed to 0, 0.5 or 1.5 mM iAs from 4–120 hpf, then it was removed, and the animals were reared according to standard aquaculture protocols (Fig. 2A). As expected, 0.5 mM exposed embryos had no obvious abnormalities while those exposed to 1.5 mM were shorter and had melanocyte clustering over the head (Fig. 2B). We investigated changes in gene expression in the liver and bulk DNA methylation immediately upon completion of iAs exposure and in subsequent time points, and evaluated survival, morphology, hepatic steatosis, liver size and reproductive success after iAs removal. We then assessed the generational response to iAs toxicity in their offspring (Fig. 2C).

**Fig. 3.**
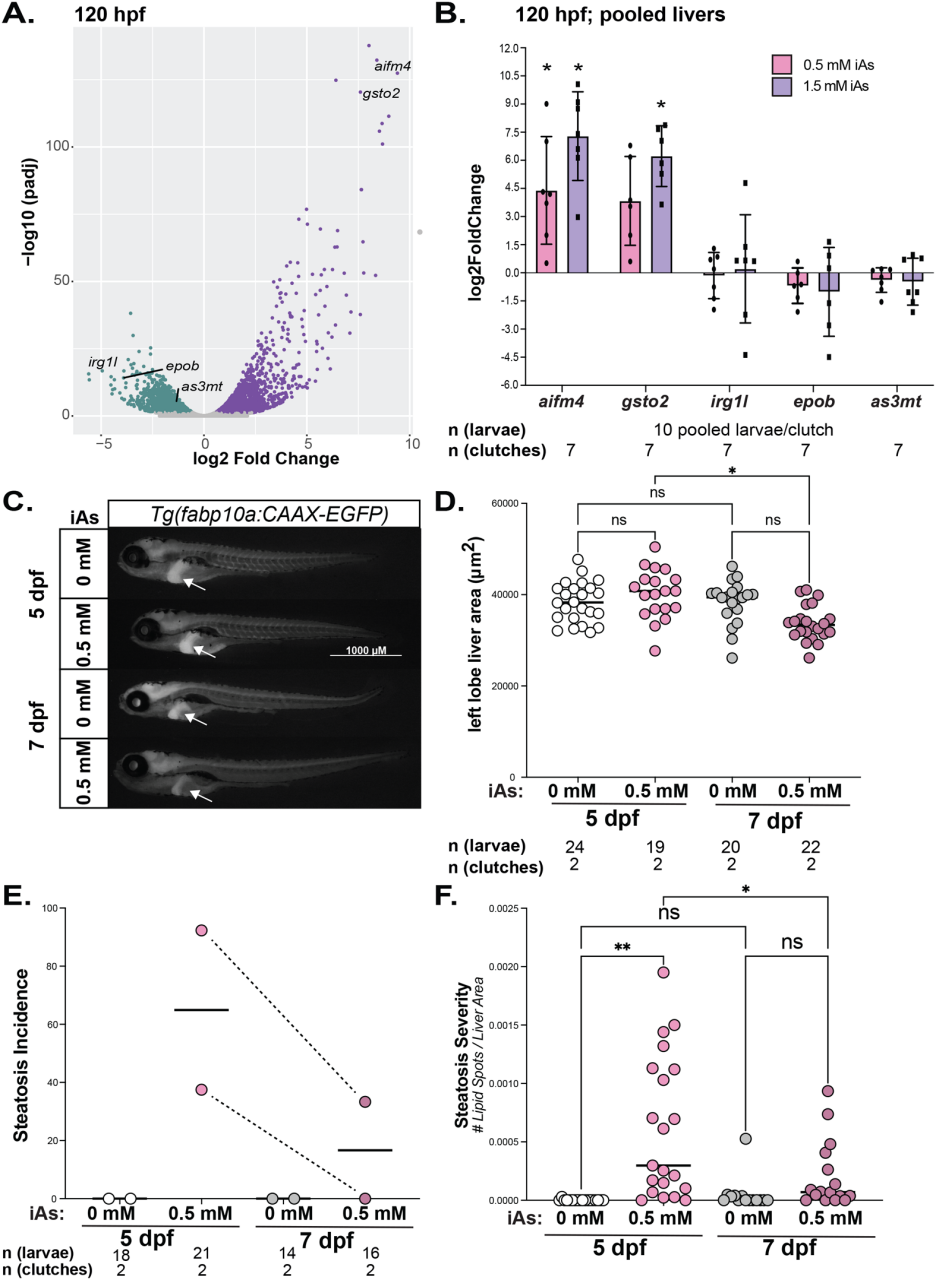
**Developmental iAs exposure induces robust gene expression changes in the liver of zebrafish, increases steatosis and reduces liver size.** (A) Volcano plot of RNA-seq data from pools of zebrafish livers following exposure to 1 mM iAs from 4–120 hpf compared to control untreated larvae. The significant (padj<0.05) downregulated genes are labelled in green and upregulated genes are labeled in purple. Selected up and down DEGs for further analysis are labelled. (B) qPCR analysis from seven clutches, with 10 livers pooled per clutch collected after 4–120 hpf exposure to 0, 0.5 and 1.5 mM iAs exposure. Values are expressed as the log2 fold change for each clutch comparing treated to untreated controls. * denotes *P*-value<0.05, Students *t*-test. Quantification of the left liver lobe area (C,D) and steatosis (E,F) of 120 hpf (5 dpf) and 168 hpf (7 dpf) larvae that were untreated or exposed to 0.5 mM iAs from 4–120 hpf. Left liver lobe area (arrow) was measured in *Tg(fabp10a:CAAX-EGFP)* transgenic larvae. Steatosis incidence was measured in Nile Red stained larvae by quantifying the number of livers with more than two lipid spots per clutch divided by the total number of larvae. Steatosis severity was measured by dividing the number of lipid spots by the liver surface area in each larvae. Scale bar: 1000 µm. the number of larvae and clutches are indicated for each condition. * and ** denote *P*-values<0.05 and <0.01 respectively, by two-way ANOVA.

To identify genes that are highly iAs-responsive in the liver, we analyzed the effects of this exposure protocol on the hepatic transcriptome at 120 hpf, immediately after exposure to 1 mM iAs, using previously published RNA-seq dataset that we generated from pools of microdissected larval zebrafish livers (Bambino et al., 2018). Nearly 23% of all genes were differentially expressed (padj<0.05) in these samples **(**Fig. 3A**)**. To determine whether exposure to lower (0.5 mM) and higher (1.5 mM) iAs concentrations from 4–120 hpf had the same transcriptomic response, we analyzed a subset of differentially expressed genes (DEGs) that participate in a wide array of hepatic functions, including xenobiotic metabolism, inflammation, and cytokine activity with no two genes overlapping in the same function. We used qRT-PCR to analyze selected upregulated [*aifm4* and *gsto2* (log2FoldChange>5, padj<0.05)] and downregulated [*irg1l* and *epob* (log2FoldChange<−3, padj<0.05)] genes (Fig. 3A) by quantitative real-time PCR in the liver of 120 hpf larvae. We also analyzed the expression of *as3mt* which metabolizes iAs (Ajees and Rosen, 2015), which we previously showed is downregulated in zebrafish livers in response to iAs exposure (Bambino et al., 2018; Delaney et al., 2020). Of these, *aifm4 and gsto2* were upregulated more in response to 1.5 mM than to 0.5 mM iAs, indicating a dose-dependent pattern (Fig. 3B).

We investigated the effects of developmental exposure to iAs on other parameters relevant to liver function: size and lipid accumulation (steatosis). While there was no effect at the end of the exposure at 120 hpf, the liver of treated larvae was significantly smaller 2 days after removal (at 7 dpf) (Fig. 3C,D). Previous studies have shown iAs exposure caused lipid accumulation in the liver. We asked whether steatosis persists after developmental exposure to 0.5 mM iAs using Nile Red to detect hepatic lipid droplets immediately after exposure terminated (at 120 hpf) and then 2 days after removal at 7 dpf (Fig. 3E,F). As we previously showed using a higher iAs concentration (Bambino et al., 2018), untreated larvae had no steatosis. Treated larvae developed steatosis at 5 dpf and by 7 dpf, steatosis reduced, although it persisted in some (Fig. 3E,F). Therefore, iAs can have a persistent, albeit modest, effect on hepatic lipid metabolism in zebrafish.

### Developmental exposure to iAs impacts survival in a dose-dependent pattern

**Fig. 4.**
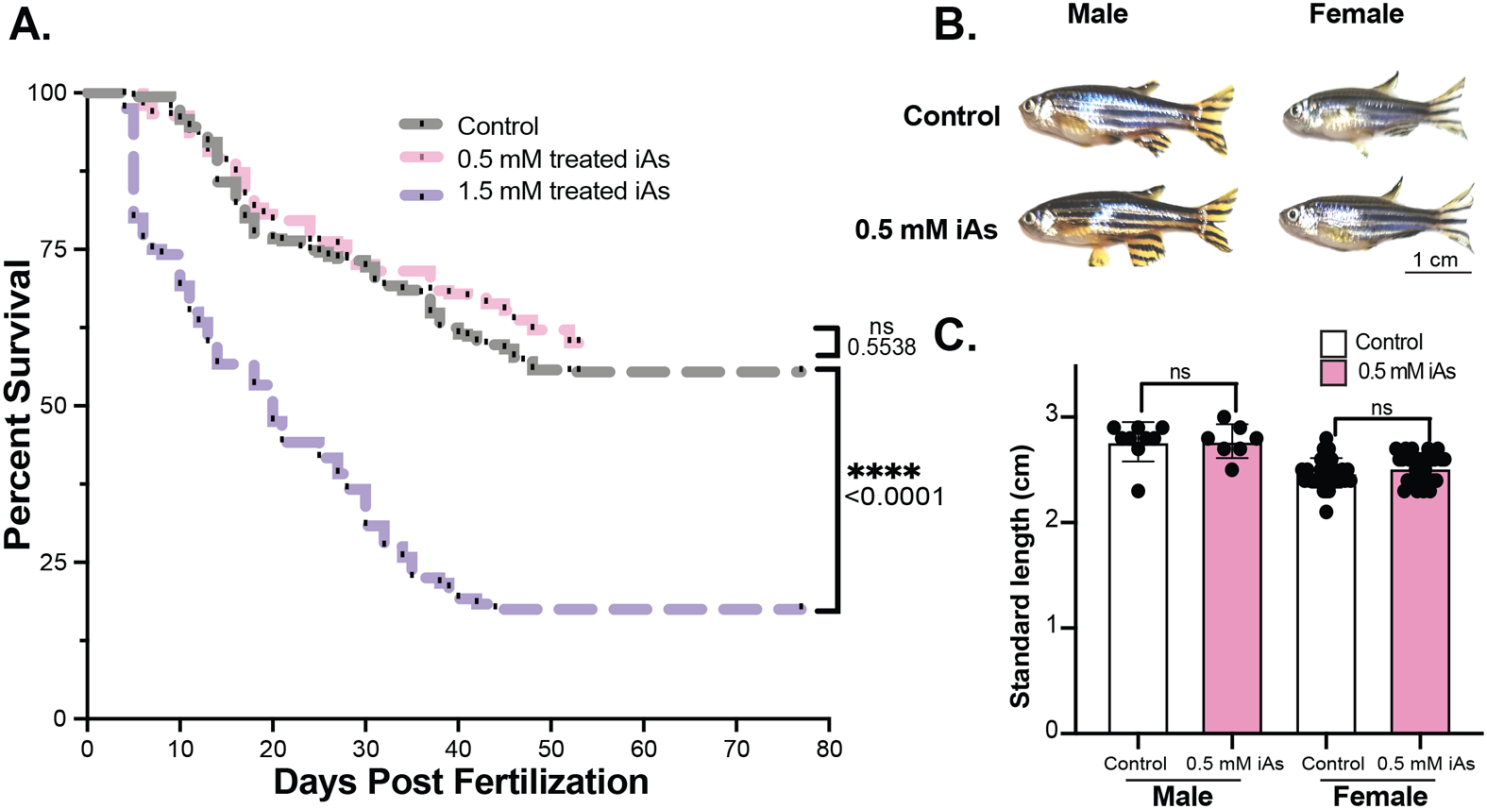
**Developmental exposure to iAs negatively impacts long-term survival but not adult morphology.** (A) Survival plot of control (grey), 4–120 hpf 0.5 mM iAs treated larvae (pink) and 4–120 hpf 1.5 mM iAs treated larvae (purple). **** denotes *P*-value<0.0001, curve comparison (Mantel–Cox). (B) Representative images of 8-month-old male and female zebrafish that were developmentally exposed to 0.5 mM iAs compared to untreated controls. Scale bar: 1 cm. (C) Standard length (cm) of 8-month-old males (left) and females (right). Not significant (ns) by Student's *t*-test.

Developmental exposure of zebrafish embryos to iAs has been shown to cause mortality in early adulthood (Abu Bakar et al., 2022). We extended this study by assessing the dose-dependent survival impact up to 3 months of age, after which other investigators found no further decreased survival in iAs treated fish. Tracking survival of zebrafish that were developmentally exposed to 0.5 and 1.5 mM iAs compared to untreated siblings throughout maturation and adulthood revealed acute mortality immediately following removal of 1.5 mM iAs, and these animals continued to exhibit increased mortality until 40 dpf, with a survival rate of 17% at 50 dpf compared to 55% for controls (Fig. 4A). In contrast, animals that were developmentally exposed to 0.5 mM iAs had no difference in survival compared to untreated controls (Fig. 4A) and they showed no changes in overall morphology (Fig. 4B) or standard length (Fig. 4C) by 8 months of age.

### Developmental exposure to iAs causes a persistent change in the *gsto2* gene

**Fig. 5.**
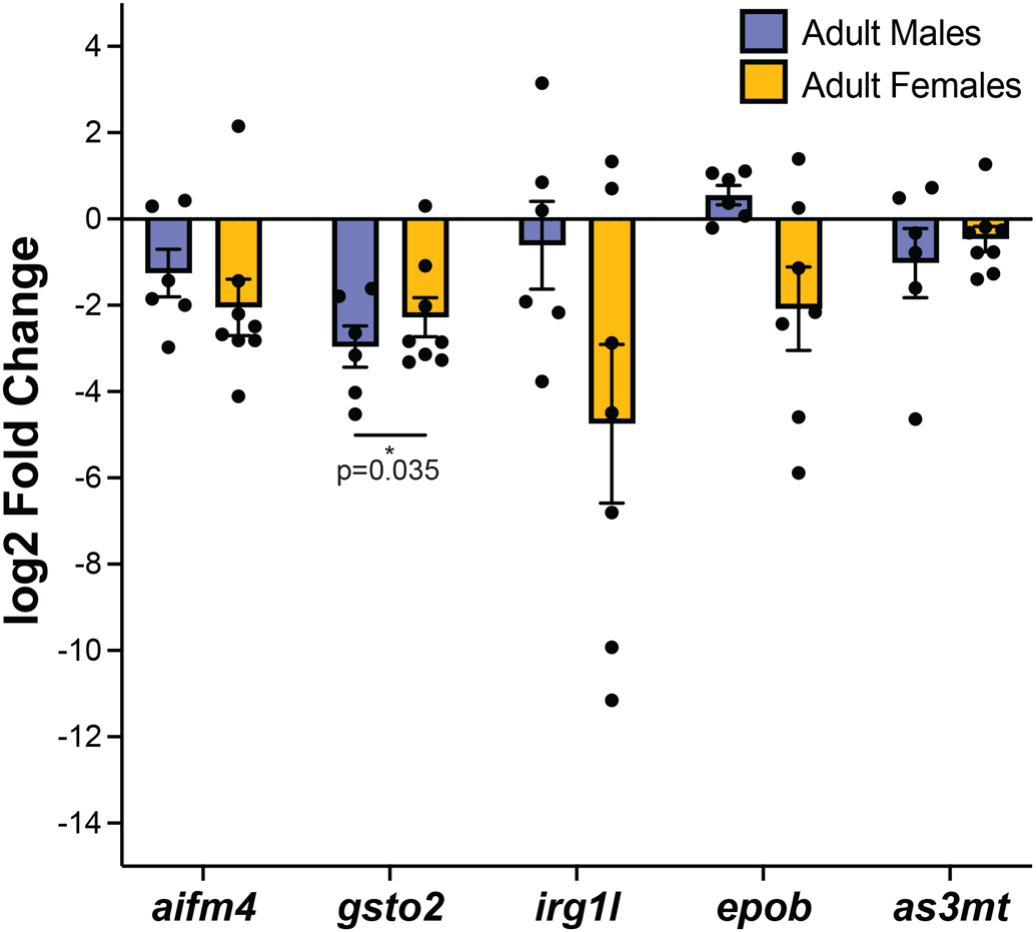
**Developmental exposure to iAs sustains differential gene expression in *gsto2* in adult livers post wash out.** qPCR data from adult male and female single livers dissected from age-matched adult zebrafish (9–17 months old). Purple bars are from males and yellow bars are from females from the 0.5 mM 4–120 hpf iAs exposed group. *n*=7, 2 clutches. Values are expressed as the log2 fold change comparing each individual treated liver to the average of control livers. * denotes *P*-value<0.05, unpaired *t*-test between untreated and iAs treated 2^deltaCT values.

Since developmental exposure to 1.5 mM iAs resulted in high mortality, we reasoned that the animals that survived to adulthood could be adapted to iAs and may not have any long-term effects. We therefore analyzed adults that were developmentally exposed to 0.5 mM iAs and used qPCR to assess the expression of some of the genes that were most significantly deregulated in the liver of 120 hpf larval zebrafish immediately following exposure (Fig. 3A,B) in adult livers of animals that were developmentally exposed and controls. RNA was extracted from the liver of seven males and seven females which were developmentally exposed to 0.5 mM iAs (ages 9–17 months) and an equal number of age matched untreated controls were assessed by qPCR for the same panel of DEGs evaluated at 5 dpf (Fig. 3B). We found that the *gsto2* gene which was significantly upregulated immediately following iAs treatment was significantly downregulated in female livers and the average fold change in male livers was reduced, but not statistically significant. Further, the average fold change of *aifm4* and *epob* was reduced, though not significant in female livers treated with iAs compared to those that were not (Fig. 5). This demonstrates that developmental iAs-exposure elicits long-term changes to *gsto2* expression in female livers, but these changes are different from the expression changes caused by immediate iAs-exposure.

### Developmental exposure to iAs does not reduce bulk DNA methylation levels

**Fig. 6.**
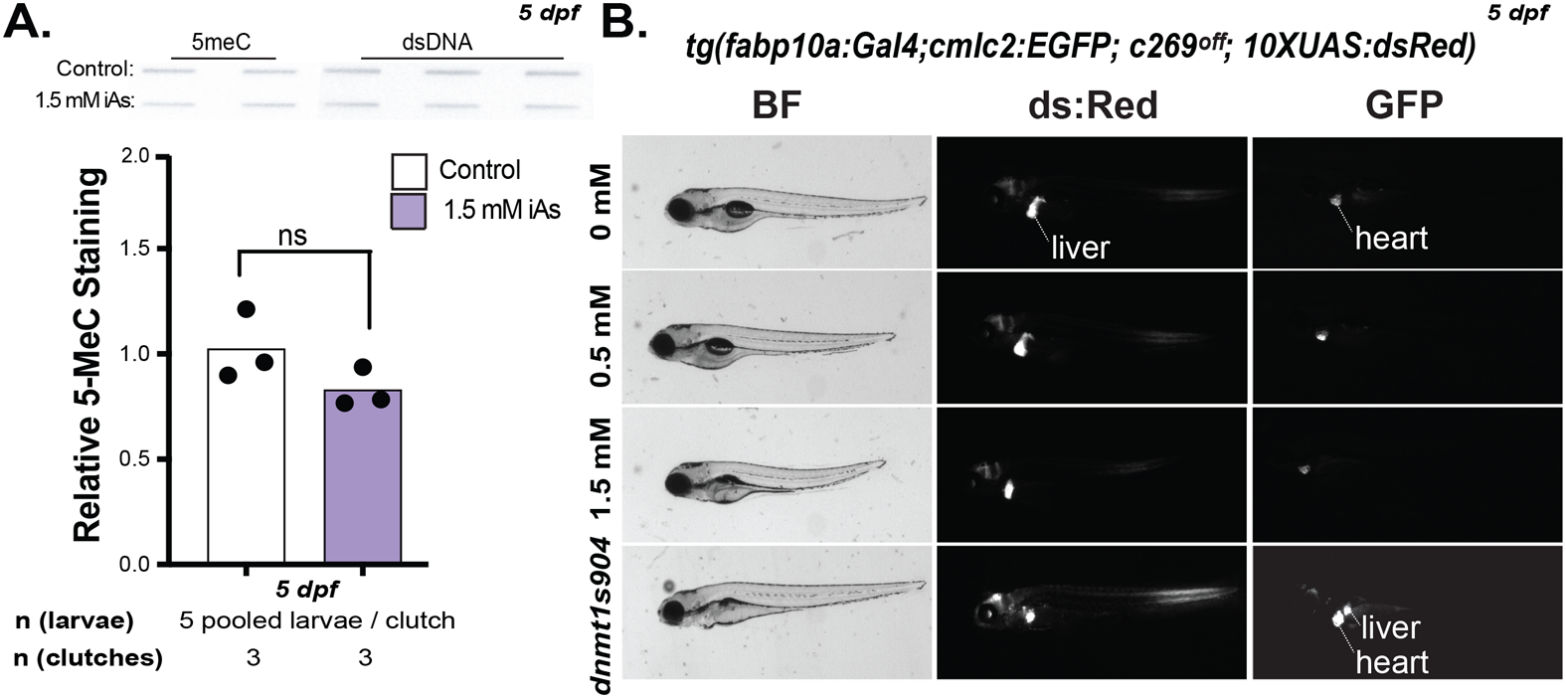
**Developmental exposure to iAs does not cause DNA hypomethylation.** (A) Slot blot hybridization from bulk DNA isolated from whole zebrafish after 0 and 1.5 mM iAs (4–120 hpf) exposure at 5 dpf (*n*=5 pooled larvae per clutch, three clutches). ns=not significant by Students *t*-test. (B) Representative brightfield and fluorescent images of 5 dpf *tg(fabp10a:Gal4;cmlc2:EGFP; c269*^o*ff*^*; 10XUAS:dsRed)* zebrafish larvae following 0, 0.5 and 1.5 mM iAs exposure from 4–120 hpf. Heart and liver are annotated. *dnmt1s904* zebrafish larvae included as a positive control for hypomethylation. dsRed in the liver indicates the presence of active Gal4 in hepatocytes; GFP in the liver indicates DNA hypomethylation.

The developmental origins of health and disease theory proposes that epigenetic changes caused by stress or toxicants during development are the mechanisms underlying the persistent effects. Some studies indicate that iAs exposure can cause DNA hypomethylation, which may contribute in part to long-term iAs-induced disease (Intarasunanont et al., 2012; Nohara et al., 2011). We used biochemical and genetic techniques to examine whether iAs caused DNA hypomethylation during exposure and after cessation. As toxicant exposures generally have very modest effects on DNA methylation levels, we used a high concentration of iAs for these studies to maximize the chance of observing an effect. Whole larvae that were exposed from 4–120 hpf to 0 or 1.5 mM iAs were collected at 120 hpf. Slot blot analysis of 5-methylcytosine (5-MeC) and double stranded DNA showed that there was no difference in the relative levels of 5-MeC (Fig. 6A). The liver is among the most sensitive target organs for iAs toxicity and since assessment of 5-MeC whole larvae may not reveal tissue specific changes we used a transgenic line that serves as a reporter of DNA methylation in live larvae (Goll et al., 2009; Magnani et al., 2021) [*tg(fabp10a:Gal4;cmlc2:EGFP; c269^off^; 10XUAS:dsRed)*]*.* This line relies on the Gal4-UAS expression system in which Gal4 targets transgenes that have the UAS promoter. In this line, the 10xUAS:GFP cassette, which is in the c269 allele is silenced by DNA methylation and only expressed in hepatocytes when the c269^off^ promoter is unmethylated and there is the presence of Gal4. This line also incorporates an unmethylated UAS driving dsRed. Therefore, all fish with Gal4 in hepatocytes will express dsRed, but only those with a 10xUAS:GFP cassette will express GFP, as shown in DNA methyltransferase (*dnmt1*) mutant larvae which serve as a positive control. No GFP was detected in the liver of iAs treated larvae even though they had active Gal4 in hepatocytes as shown by dsRed expression (Fig. 6B). This demonstrates that iAs does not reduce the level of DNA methylation in the zebrafish liver to an extent that is sufficient to activate this transgenic reporter.

### Developmental exposure to iAs reduces mating success in adults

**Fig. 7.**
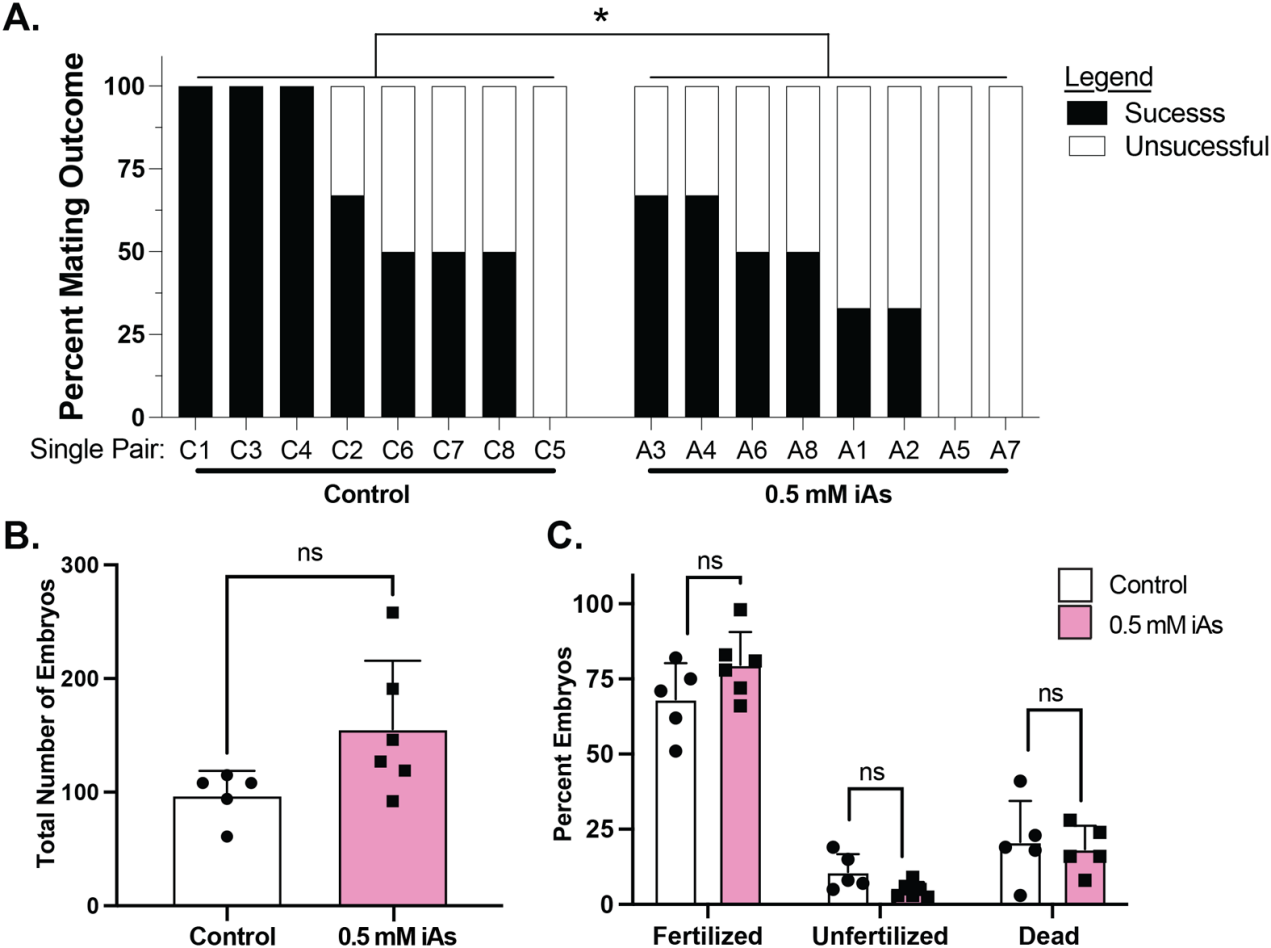
**Developmental iAs exposure reduces mating success but does not affect the number of fertilized embryos.** (A) Percent of mating success between eight single pairs of zebrafish that developed from untreated or 0.5 mM iAs developmentally treated zebrafish. *P-value<0.05 by Chi Square. (B) The number of embryos produced from successful matings of control and 0.5 mM iAs developmentally treated zebrafish. (C) Percent of embryos that were fertilized, unfertilized, and dead from successful mating of control and 0.5 mM iAs developmentally exposed zebrafish. ns: not significant, Students T-test.

To evaluate whether developmental exposure to iAs impacts mating success, the number of spawned eggs and fertilization rate were assessed for eight pairs of males and females that were reared from developmentally exposed (0.5 mM iAs) or unexposed control embryos. The number of times each pair produced offspring was scored as negative for no embryos and positive for one or more embryos produced and each pair was mated up to five times. Nearly all pairs of control animals (7/8) mated at least 50% of the time, with three pairs producing embryos in all five mating trials. The mating success was significantly lower for pairs that were developmentally exposed to 0.5 mM iAs: two of the eight pairs never mated, and of those that did mate, none were successful in all trials. (Fig. 7A). Of those pairs that did produce offspring, we observed no significant difference in the number of eggs, fertilization rate or developmental progress between embryos generated from control or iAs exposed pairs (Fig. 7B,C). A similar pattern was observed for offspring generated from adults developmentally exposed to 1.5 mM iAs (Fig. S1A,B).

### iAs toxicity is not changed in offspring from developmentally exposed parents

**Fig. 8.**
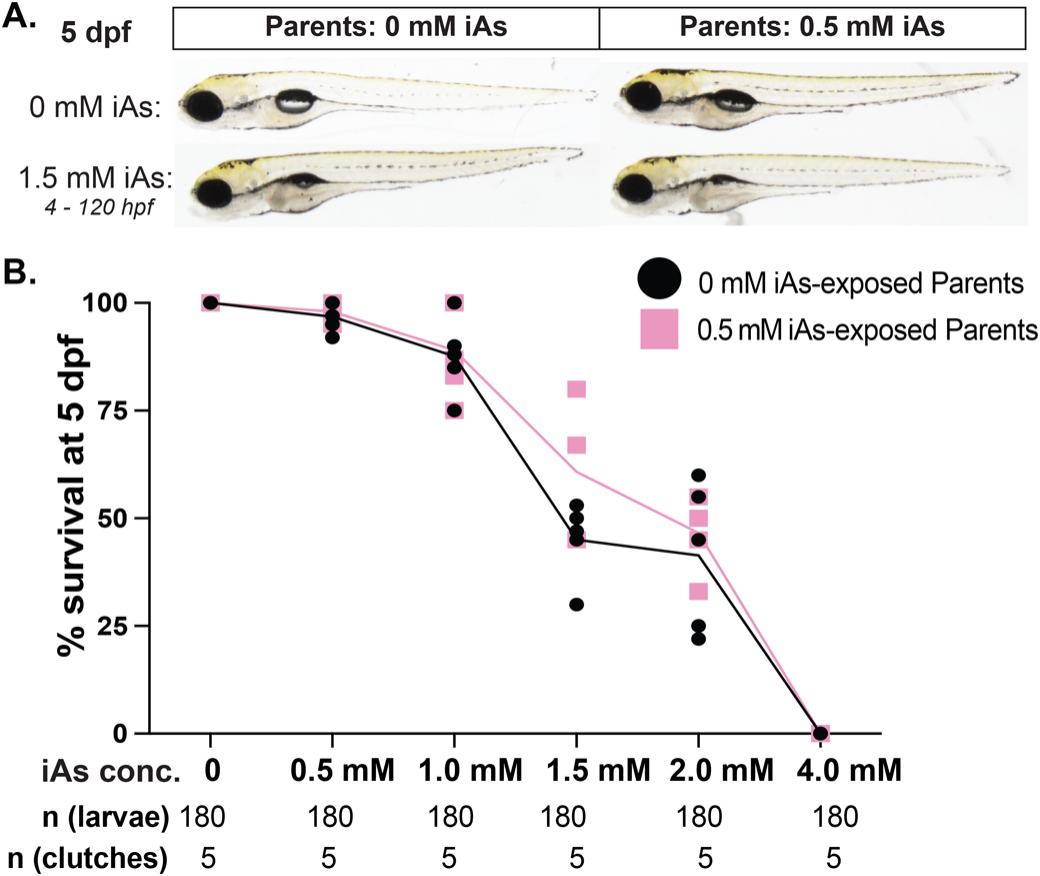
**Parental development exposure to iAs does not alter the toxicity of iAs in offspring.** (A) Representative images of 5 dpf F1 larvae produced from incrosses of untreated parents (left) and parents that were developmentally exposed to 0.5 mM iAs (right). F1 were treated to 0 (top) and 1.5 mM iAs from 4–120 hpf (bottom). (B) Survival curve of F1 produced from incrossing parents exposed to 0 (black) or 0.5 mM iAs from 0–120 hpf (pink) after 4–120 hpf exposure to 0, 0.5, 1.0, 1.5, 2.0 and 4.0 mM iAs (*n*=20 larvae per clutch per group, five clutches).

To determine if parental exposure to iAs during development (F0) alters iAs toxicity in offspring (F1), we exposed F1 embryos generated from parents that were developmentally exposed to 0.5 or 1.5 mM iAs to a range of iAs concentrations from 4–120 hpf and scored for mortality. There was no difference in the LC_50_ of iAs or the morphology of larvae from control or iAs exposed parents (Fig. 8A,B; Fig. S1C). Thus, while developmental exposure of zebrafish reduces mating success, the offspring do not have any overt developmental defects or changes in response to iAs.

## DISCUSSION

The developmental origins of health and disease field has generated a wealth of evidence that early life toxicant exposure leads to poor health outcomes later in life, and some of these adverse outcomes are passed to offspring (Nicolella and de Assis, 2022). Toxicants like iAs can cause disease long after the exposure has been terminated. Several studies have investigated the effects of developmental iAs exposure on zebrafish behavioral and neurological parameters, but none have investigated the effects on the liver. We used zebrafish to investigate how developmental exposure to iAs causes sustained effects on the liver, on reproductive outcomes and generational susceptibility to iAs. We report that the expression of *aifm4* and *gsto2* were altered following developmental exposure, and that *gsto2* was significantly downregulated in female adult livers months after iAs was removed. Epigenetic modifications, including DNA methylation, have been proposed as a mechanism of long-term and generational effects of developmental exposures, but we found no effect on bulk DNA methylation levels in whole animals or the liver. We report that a high concentration of iAs during development caused acute mortality immediately after removal and increased mortality persisted through adulthood, while lower concentrations of iAs were well tolerated during development and did not affect survival but did result in effects on liver size, persistent steatosis, and, in adults, a significant decrease in mating success. This data narrows the focus to those aspects of fish biology that are impacted by developmental exposure to iAs to hepatic *gsto2* expression changes and reproductive success.

The liver carries out a wide variety of biological functions, including energy metabolism through homeostasis of glucose and lipids, protein metabolism, production of bile and serum proteins and metabolism of urea and toxins. Toxins cause hepatocellular injury and cell death and the regenerative capacity of the liver can replace lost or damaged tissue. Here, we show that acute (96–120 hpf) iAs exposure causes a decrease in liver enriched gene expression immediately after washout at 120 hpf, suggesting that iAs causes liver damage, but that this damage can recover within a week following iAs removal. Notably, at 2 days post iAs washout, expression of *fabp10a* is strongly upregulated, which could be explained by transcriptional adaptation in which hepatocytes upregulate important functional genes to maintain essential liver processes to compensate for damaged or dividing cells. This has been shown in the liver following acetaminophen injury (Walesky et al., 2020) and future studies are needed to determine whether transcriptional compensation sustains liver function during iAs-induced liver injury and recovery.

Our finding that iAs induced long-term changes in *gsto2* expression in the liver are consistent with other studies demonstrating that developmental exposure to iAs causes changes in gene expression patterns related to neurological disorders. In zebrafish, developmental exposure to iAs (5–72 hpf) caused changes in the expression of genes implicated in autism spectrum disorders at 6 dpf, though only two were significantly upregulated and one was downregulated and it is unknown if this pattern was retained in adulthood (Abu Bakar et al., 2022). Another study showed that the brain-derived neurotrophic factor (*bdnf*) gene which is implicated in neuronal growth was downregulated in F2 offspring generated from animals that were exposed to iAs from 4 hpf–150 dpf (Valles et al., 2020). A study in mice showed that pregnant dams fed iAs through drinking water resulted in decreased expression in F2 offspring in some genes, including *BDNF* (Htway et al., 2021). This suggests iAs exposure as a cause of neurological defects in exposed animals and their offspring. Follow-up investigations are necessary to determine whether neurological or behavioral defects could alter behaviors required for successful mating and accounts for the reduced mating success in adults that were exposed to iAs during development.

No studies to date have investigated the persistent effects of iAs on the liver in zebrafish. We identified reduced liver size and steatosis to be a persistent effect of iAs after 2 days of recovery, but have not yet established whether this persists to later stages. Gene expression analysis of a subset of genes that are highly iAs responsive in the liver was performed. Interestingly, genes that were highly upregulated after immediate iAs exposure were downregulated in adulthood, suggesting a rebound effect on their expression. GSTO2 can conjugate GSH to certain environmental toxins, like iAs, rendering them less toxic and facilitating elimination (Paiva et al., 2010). This is significant, as human studies have shown that alleles that decrease GSTO2 function are associated with an increased risk of skin lesions caused by iAs exposure (Luo et al., 2018). This suggests that developmental exposure to iAs could increase susceptibility to iAs toxicity in adulthood. Similarly, *aifm4*, which is predicted to regulate mitochondrial functions, may have an adaptive effect during immediate iAs challenge but become maladaptive over prolonged exposure, potentially explaining the immediate high expression following iAs exposure followed by a return to baseline expression in adulthood.

Our finding that developmental iAs did not reduce bulk DNA methylation immediately after exposure or later after recovery is inconsistent with some studies that suggest DNA hypomethylation as a mechanism of long-term iAs-toxicity (Nohara et al., 2011; Okamura et al., 2019; Reichard et al., 2007). However, the different approaches used to assess DNA methylation changes are an important consideration for these conflicting results. For instance, a recent study using zebrafish embryos reported that developmental exposure to iAs caused an 87% reduction in global DNA methylation (Silva et al., 2023), when in fact the assay used in these experiments measured 5-hydroxymethyl cytosine, not 5-mC, and therefore the conclusions from this study are questionable. While our data indicate that the transcriptomic changes observed in response to iAs exposure are not attributed to bulk DNA hypomethylation, it is possible that locus specific changes or other epigenetic modifications occur in response to iAs exposure.

Finally, our finding of reduced mating success in adults that were exposed to iAs during development has implications for the fish populations exposed to iAs in their environment. Additionally, this data expands on the studies on iAs exposure and reproductive health outcomes. Developmental iAs exposure in rodents has significant negative effects on sperm quality and quantity (Kim and Kim, 2015; Lima et al., 2018; Pant et al., 2001) and iAs exposure in women has been linked to a wide range of reproductive health issues (Malin Igra et al., 2023; Rahman et al., 2017; Rahman et al., 2021; Tofail et al., 2009). This study provides a model to investigate the mechanism of iAs-induced reproductive outcomes, although it is unclear to what extent the mating success decline in iAs exposed animals can be attributed to defects in gametogenesis or to behavior. Future investigations into whether the reduction in mating success is attributed to male, female or both sexes in zebrafish will help identify groups that are most at risk to long-term impacts in fertility.

## MATERIALS & METHODS

### Zebrafish husbandry and embryo rearing

All procedures were approved by and performed in accordance with the New York University Abu Dhabi Institutional Animal Care and Use Committee (IACUC) guidelines. Adult wild type (WT; ABNYU, TAB5, and BNY14) and transgenic lines [*Tg(fabp10a:Gal4;cmlc2:EGFP; c269*^o*ff*^*; 10XUAS:dsRed)*] were maintained on a 14:10 h light:dark cycle at 28°C. All experiments were conducted in six-well plates (Corning, NY, USA) with 20 embryos in 10 ml embryo medium, which is prepared in accordance with the Zebrafish Information Network protocol (Westerfield, 2007). Embryos were collected from group matings or single mating pairs, as indicated, within 2 h of spawning and were reared at 28°C, according to standard conditions.

### iAs exposure in embryos and adult rearing

Embryos were exposed to sodium meta-arsenite (Sigma-Aldrich, MA, USA; henceforth referred to as iAs) by diluting 0.05 M stock solution to final concentrations ranging from 0.5 mM–4 mM in embryo medium from either 4–120 hpf or 96–120 hpf, as indicated in the text. The iAs stock is aqueous and diluted in embryo medium and therefore as a control, all comparisons are to untreated siblings. A detailed overview of how treatment parameters were optimized are previously described (Ramdas Nair et al., 2021). For all experiments, mortality was scored daily, dead embryos and larvae were removed, and morphological parameters were recorded at 120 hpf.

After 120 hpf, all treated larvae were washed three times in fresh embryo media prior to placing on the NYUAD Fish Facility Aquaculture system (Techniplast) alongside untreated control siblings.

### Image acquisition

For whole mount imaging of live larvae, embryos were anesthetized with 500 µM tricaine (Ethyl 3-aminobenzoate methanesulfonate; Sigma-Aldrich), mounted in 3% methylcellulose on a glass slide and imaged on a Nikon SMZ25 stereomicroscope.

### Liver size measurement

To quantify liver size, images were acquired at two time points including 120 hpf and 168 hpf and assessed as described (Magnani et al., 2024). In brief, transgenic embryos *tg(fabp10a:CAAX-EGFP)* were exposed to iAs from 4–120 hpf, and half the clutch was anesthetized at 120 hpf 500 μM of tricaine (Ethyl 3-aminobenzoate methanesulfonate; Sigma-Aldrich) and placed in 3% methylcellulose and imaged on Nikon SMZ25 stereomicroscope using the GFP filter at 2× magnification and 125 ms resolution and 1.40× gain. The other half of the clutch was cultured in the aquaculture system, fed paramecium ad labium and collected at 168 hpf and imaged using the same approach. The area of the left liver lobe was highlighted manually through the drawing tool on Image J. Liver size measurements were conducted on 8–10 larvae for each treatment condition in two clutches.

### Steatosis assessment

To measure lipid accumulation for steatosis assessment, larval samples at 119 hpf were treated with 500 ng/ml Nile Red for 1 h, fixed in 4% Paraformaldehyde (UTECH) for 4–24 h and washed with 1×PBS (UTECH) before imaging. All samples were embedded using 1% low melting agarose (SeaPlaque Agarose, Lonza) and were imaged on an inverted confocal microscope (Leica STED 3×) using a 63× water objective lens. Larvae were scored as positive for steatosis if they had more than two lipid droplets per liver and steatosis incidence was calculated as the percent of larvae per clutch scored as positive. Steatosis severity was assessed using Imaris software to spot count the number of lipid droplets per total liver section area.

### Gene expression analysis

Pools of at least five livers were microdissected from 120 hpf zebrafish larvae with transgenic marked livers [*Tg(fabp10a:CAAX-eGFP)*]. Larvae were anesthetized in tricaine and immobilized in 3% methyl cellulose and the livers were removed using 30-gauge needles. RNA was extracted from livers using TRIzol (ThermoFisher Scientific, 15596026) and precipitated with isopropanol as described (Vacaru et al., 2014). RNA was reverse transcribed with qScript (QuantaBio, 95048-025).

Gene expression was assessed using RNA-seq or quantitative reverse transcription PCR (qRT-PCR). qRT-PCR was performed using Maxima Sybr Green/ROX qPCR Master Mix Super Mix (ThermoFisher Scientific, K0221). Samples were run in triplicate on QuantStudio 5 (ThermoFisher Scientific). Target gene expression was normalized to *ribosomal protein large P0* (*rplp0*) using the comparative threshold cycle (ΔΔCt) method (Livak and Schmittgen, 2001). Primers for the genes of interest are listed in Table S2. Expression in treated animals was compared to untreated controls from the same clutch to determine fold change.

### RNA-seq

The publicly available RNA-seq dataset was generated previously in the Sadler Lab (GSE104953) from microdissected livers from untreated (control) or exposed to 1 mM iAs from 4–120 hpf transgenic [*tg(fabp10a:nls-mcherry)*] zebrafish larvae (Bambino et al., 2018) and reanalyzed. To determine differential expression, the DESEQ2 package (Love et al., 2014) was used for the analysis of the raw count data, previously generated by HTSEQ count (normalization and testing for differential gene expression analysis), and the default implementation of DESEQ2 was used. By default, *P*-values in DESEQ2 are computed using the Wald test and corrected for multiple testing using the Benjamini and Hochberg (BH) method. The BH implementation in DESEQ2 controls the false discovery rate (FDR) by first ranking all the genes according to their *P*-value, and subsequently multiplying each ranked *P*-value by m/rank. An FDR threshold is then set, using 0.05 as a cutoff. These adjusted *P*-values were used to determine differentially expressed genes (padj<0.05). Volcano plots were plotted using R package ggplot2.

### Slot blot

Slot blot was performed using 0.5 ng of gDNA from pools of five 5 dpf or three 21 dpf zebrafish larvae. gDNA was denatured in 400 mM NaOH/10 mM EDTA and blotted onto nitrocellulose membrane (BioRad) in duplicate for 5 mC DNA and triplicate for dsDNA using a slot blot apparatus (BioRad). Equivalent volume of DNAse/RNAse-free water (Invitrogen) was loaded instead of genomic DNA as negative control (data not shown). Membranes were incubated 1 h at 80°C, blocked with 5% bovine serum albumin (BSA) in TBST (37 mM NaCl, 20 mM Tris pH 7.5, 0.1% Tween 20), and incubated overnight at 4°C in either anti-dsDNA (Abcam, 1:5000 in 2% BSA in TBST) or anti-5-methyl-cytosine (5 mC; Aviva Biosystem clone 33D3, 1:3000 in 2% BSA in TBST). Membranes were washed in TBST and probed with anti-mouse HRP secondary antibody (Promega; 1:2000 in 5% BSA in TBST) for 1 h at room temperature followed by development in ECL (ThermoFisher Scientific) or Clarity ECL (BioRad). ChemiDoc (BioRad) was used to detect and quantify the chemiluminescent signal. Gel Analyzer (http://www.gelanalyzer.com) was used to perform quantitative densitometric analysis of the signals and ratio between 5 mC and dsDNA was plotted for each sample using GraphPad Prism.

### Statistical analysis, rigor, and reproducibility

Experiments were carried out on at least 2–3 clutches of embryos in most cases and at least three adult animals, with all replicates indicated, unless otherwise specified. Reproducibility was assured by carrying out key experiments, including phenotype scoring by independent investigators. Data are presented as normalized values. Statistical tests were used as appropriate to the specific analysis, including Student's *t*-test, Chi Square (Fisher's exact test), two-way analysis of variance (ANOVA) and curve comparison (Mantel–Cox) using Graphpad Prism Software.

## Supplementary Material

10.1242/biolopen.060094_sup1 Supplementary information

Table S1.

## Diversity and inclusion statement

This work represents a collaborative effort between all authors. Some of the work was from the senior capstone project of an undergraduate student at NYUAD (A.A.K.) and a doctoral student (P.D.). The authors represent diverse identities, with differences in countries and cultures of origin (Ghana, UAE, USA,), age, religion, except they are all women.
