# Supplementary material for: Sustained effects of developmental exposure to inorganic arsenic on hepatic gsto2 expression and mating success in zebrafish
Source: Biol Open. 2024 Mar 6;13(3):bio060094. doi: 10.1242/bio.060094 (PMC10941348; doi:10.1242/bio.060094)
Supplement: Supplementary information [file biolopen-13-060094-s1.pdf]

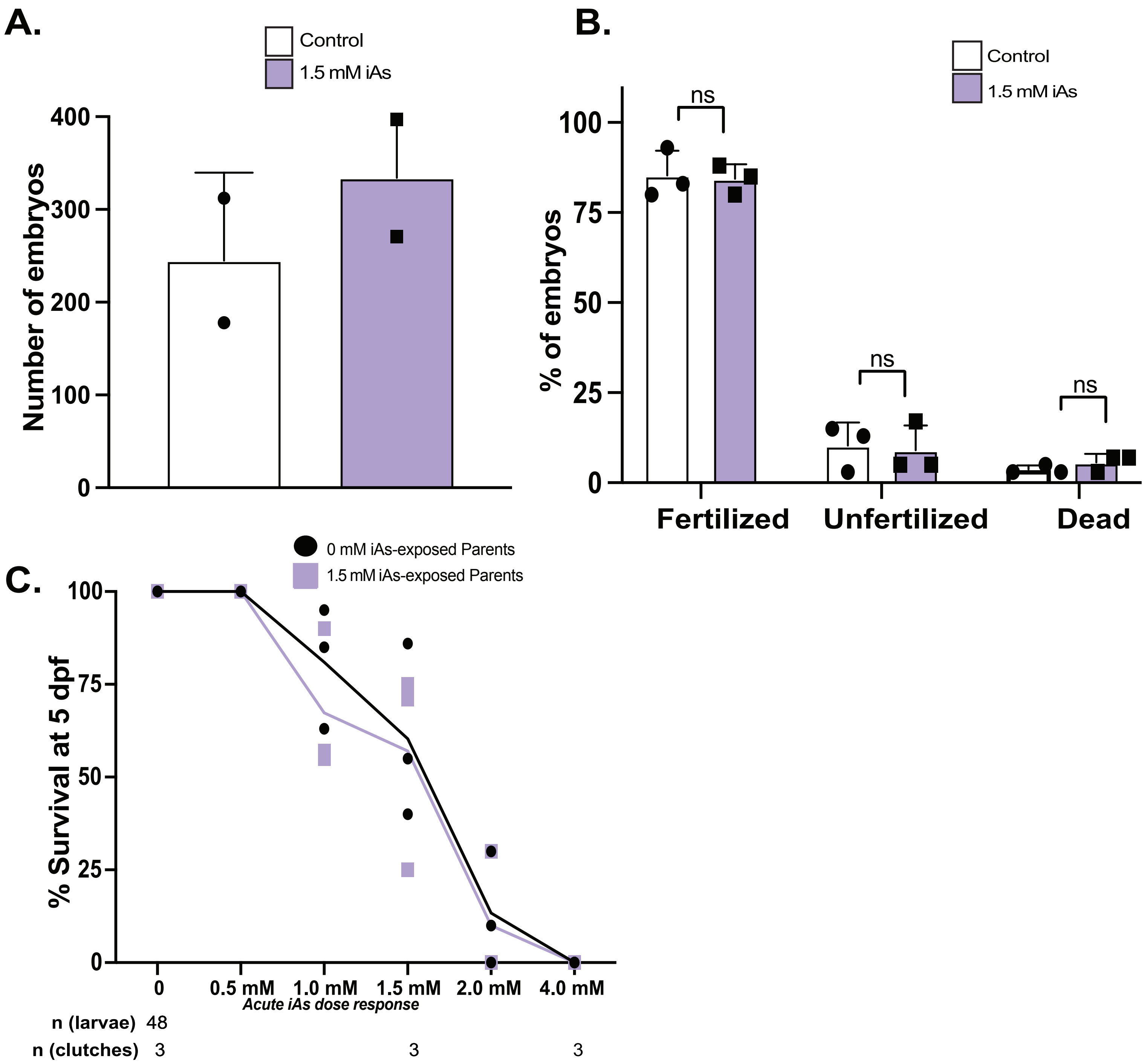
A.
Control
1.5 mM iAs
Number of embryos
400
300
200
100
0
B.
Control
1.5 mM iAs
% of embryos
100
75
50
25
0
ns
ns
ns
Fertilized
Unfertilized
Dead
C.
0 mM iAs-exposed Parents
1.5 mM iAs-exposed Parents
% Survival at 5 dpf
100
75
50
25
0
0
0.5 mM
1.0 mM
1.5 mM
2.0 mM
4.0 mM
Acute iAs dose response
n (larvae) 48
n (clutches) 3
3
3

Fig. S1.

**Table S1.**

Available for download at
https://journals.biologists.com/bio/article-lookup/doi/10.1242/bio.060094#supplementary-data

**Table S2. qPCR Primers**

| Ensembl ID | Symbol | Forward 5'-3' | Reverse 5'-3' |
|---|---|---|---|
| ENSDARG00000051783 | *rplp0* | CTGAACATCTCGCCCTTCTC | TAGCCGATCTGCAGACACAC |
| ENSDARG00000061634 | *aifm4* | CGGAGGATTTTATCTGGACAGC | CCAGAACCTCCTTCATTTGTCC |
| ENSDARG00000033285 | *gsto2* | CGAGGCTTTGGCAAACAAGAA | CGTCTTGGCCAGACAATGCT |
| ENSDARG00000062788 | *irg1l* | GCATTGGAGTTGGGCTTGTG | CAGAAGCATACATGTGCTGGC |
| ENSDARG00000100737 | *epob* | GGCACTTAATACGGCCAGGA | GGACTCGGCCTGCTGTAAAT |
| ENSDARG00000027572 | *as3mt* | CCGGGCTGGAGGATAAATCA | CCCTCCGTCCTTCAGAACAC |
| ENSDARG00000030872 | *cetp* | TTGTCGCTCTGGTCAAGATAAG | CAAGACTACAGCAGCAGGATAG |
| ENSDARG00000010312 | *cp* | AGGCAACGAGTTCCTCAAGA | GGGTCCCATTATTCCCAAGT |
| ENSDARG00000063014 | *dbpa* | GCCAGATGAACAGAAGGATGAC | CTGCTCGCACTGAAATCTGG |
| ENSDARG00000038439 | *fabp10a* | CCACCATGGACGGCAAGAAG | CCTTGATCTCCTGGATGTGGG |
| ENSDARG00000008969 | *fgb* | AGAAAGTCAGCGAGGGCAAT | ATGTTCTGGGGGAAGGTGAC |
| ENSDARG00000037281 | *fgg* | GTACTTCAGAGGAGGCGGGA | ACTCAGTCCTGTCATCGGGT |
| ENSDARG00000087697 | *lpl* | AGCCGCAAAAACCAGAGATTG | CCAGCGGAGGTGGATTTTGA |
| ENSDARG00000013561 | *pgm1* | TGCCAGATGAACAGAAGGATGAC | CTGCTCGCACTGAAATCTGG |
| ENSDARG00000013430 | *bhmt* | TATGTGAAGGCCGGACCCTG | AACGTCTGCATGACATTTGAGC |
